# Recognition memory reconsolidation requires hippocampal Zif268

**DOI:** 10.1038/s41598-019-53005-8

**Published:** 2019-11-12

**Authors:** Maria Carolina Gonzalez, Janine I. Rossato, Andressa Radiske, Marina Pádua Reis, Martín Cammarota

**Affiliations:** 1 Memory Research Laboratory, Brain Institute, Federal University of Rio Grande do Norte, Av. Nascimento de Castro 2155, RN 59056-450 Natal, Brazil; 2 Departament of Physiology, Federal University of Rio Grande do Norte, Av. Sen. Salgado Filho 3000, RN 59064-741 Natal, Brazil

**Keywords:** Neurophysiology, Learning and memory

## Abstract

Object recognition memory (ORM) serves to distinguish familiar items from novel ones. Reconsolidation is the process by which active memories are updated. The hippocampus is engaged in ORM reconsolidation through a mechanism involving induction of long-term potentiation (LTP). The transcription factor Zif268 is essential for hippocampal LTP maintenance and has been frequently associated with memory processes. However, its possible involvement in ORM reconsolidation has not been determined conclusively. Using Zif268 antisense oligonucleotides in combination with behavioural, biochemical and electrophysiological tools in rats, we found that hippocampal Zif268 is necessary to update ORM through reconsolidation but not to retrieve it or keep it stored. Our results also suggest that knocking down hippocampal Zif268 during ORM reconsolidation deletes the active recognition memory trace.

## Introduction

Object recognition memory (ORM) is a major component of declarative memory that allows animals to distinguish between novel and familiar items. ORM consolidation requires the functional integrity of several brain structures, including the hippocampus^1-5^, but see also^6^. The hippocampus is also engaged in ORM reconsolidation, a protein synthesis-dependent process that restabilizes consolidated memories weakened by retrieval. However, this only happens when ORM reactivation occurs simultaneously with novelty detection^7,8^. Remarkably, when induced by presentation of a novel object, ORM reconsolidation mediates incorporation of information concerning that object into the original memory trace through a mechanism involving hippocampal LTP induction and BDNF/PKMζ-dependent AMPAR translocation to the synaptic membrane^9,10^.

Zif268 is a member of the Egr family of zinc finger transcription factors that binds to GC-rich response elements in the promoter region of target late-response genes to regulate their expression^11^. In the brain, Zif268 is transiently expressed in response to several stimuli^12–14^. In particular, hippocampal Zif268 increases in an NMDAR-dependent manner after LTP induction^15–17^ and has been repeatedly linked to memory processing^18–20^. However, despite the fact that many consider Zif268 a non-declarative memory reconsolidation marker^20–24^, it was recently suggested that, on the contrary, Zif268 restricts the extinction of such memories^25^.

Experimental evidence linking Zif268 to declarative memory reconsolidation is scarcer. In this regard, it has been reported that delayed reexposure to familiar objects causes ORM amnesia in Zif268 knockout mice^17^. However, these mutants were unable to exhibit late LTP or form stable hippocampus-dependent long-lasting memories^21^, and had to be submitted to several learning trials to acquire an ORM trace they could remember for 48 h^20^. Therefore, whether hippocampal Zif268 is actually involved in ORM reconsolidation is an unsolved question. Here, we report that inactive ORM does not require hippocampal Zif268 to persist but becomes vulnerable to Zif268 antisense oligonucleotides (ASO) when reactivated in the presence of a novel object. A brief reminder trial able to restore a decayed ORM representation did not reverse the amnesia caused by ASO. We also found that consolidation inhibitors given upon retraining impaired ORM reacquisition in animals rendered amnesic with ASO, as if these animals had to consolidate the disrupted ORM trace anew. When taken together with findings showing that ORM reactivation in the presence of a novel object increases Zif268 hippocampal levels, our results indicate that this transcription factor is required to update ORM through reconsolidation and suggest that the amnesia caused by inhibition of this process with ASO is due to storage failure.

## Results

**Figure 1:**
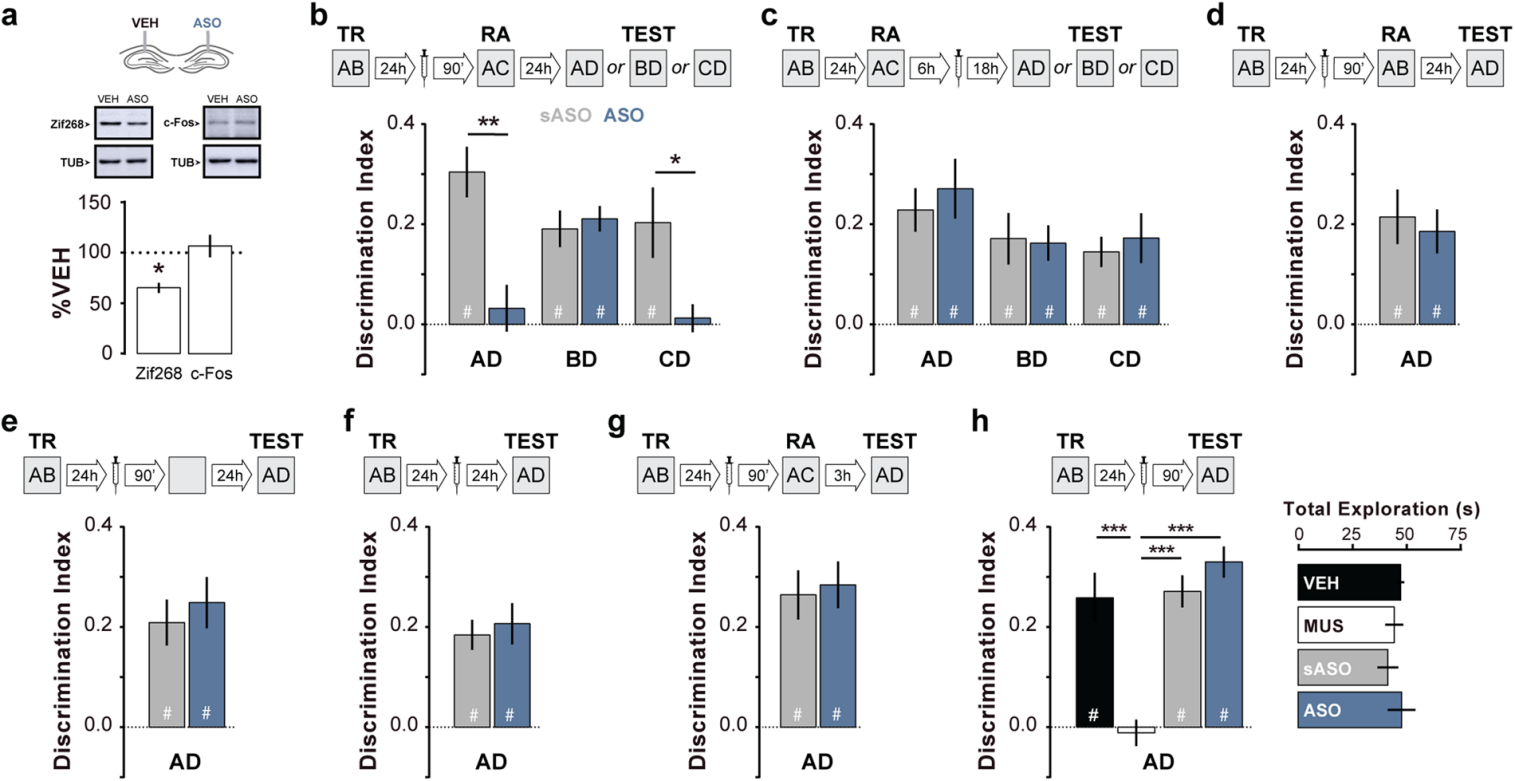
Zif268 knockdown impairs ORM reconsolidation. (**a**) Rats received vehicle (VEH; saline 0.9%) in the dorsal CA1 region of one hemisphere and Zif268 antisense oligonucleotides (ASO; 2 nmol/µl) in the other. Ninety minutes later the animals were decapitated and the dorsal CA1 region dissected out and homogenized to determine Zif268, c-Fos and β-tubulin (TUB) by immunoblot. Data are presented as mean ± SEM; n = 3 animals. For full-length blots see Supplementary Figure S1 in Supplementary Information. (**b**) Rats trained in NOR (TR) using objects A and B received bilateral intra-CA1 infusions of ASO or scrambled ASO (sASO; 2 nmol/µl) 24 h post-training. Ninety minutes thereafter, the animals were submitted to a 5′-long ORM reactivation session (RA) in the presence of familiar object A and novel object C. Retention was assessed 24 h later (TEST) in the presence of familiar objects A, B or C and novel object D. (**c**) Rats were treated as in (**b**) except that sASO and ASO were administered 6 h post-RA. (**d**) Rats were treated as in (**b)**, except that RA occurred in the presence of familiar objects A and B. (**e**) Rats were treated as in (**b**) except that sASO and ASO were given before a 5′-long exploration session of the training arena devoid of objects. (**f**) Rats were treated as in (**b**) except that RA was omitted. (**g**) Rats were treated as in (**b**) except that TEST was performed 3 h post-RA. (**h**) Rats were trained in NOR as in (**b**) and 24 h later received bilateral intra-CA1 infusions of VEH, muscimol (MUS; 0.1 µg/side), sASO or ASO. Retention was assessed in the presence of familiar object A and novel object D 90′ post-infusions. Behavioural data (mean ± SEM) are presented as discrimination index or total object exploration time during TEST; n = 7–10 animals per group. Dashed lines represent chance level. #p < 0.05 in one-sample Student’s t-test with theoretical mean = 0; *p < 0.05, **p < 0.01, ***p < 0.001 in one-sample Student’s t-test with theoretical mean = 100, unpaired t-test or Bonferroni’s multiple-comparison test after one-way ANOVA. Animals explored objects equally during TR. Total object exploration time did not differ among TR, RA and TEST. For mean object exploration time and discrimination index values during TR see Supplementary Table S1 in Supplementary Information. For mean object exploration time and discrimination index values during RA see Supplementary Table S2 in Supplementary Information. For mean object exploration time values during TEST see Supplementary Table S3 in Supplementary Information.

To study the role of hippocampal Zif268 in ORM reconsolidation we trained adult male Wistar rats in a novel object recognition-learning task (NOR) involving exploration of two different but behaviourally equivalent novel stimuli objects (A and B) in a familiar open field arena^10^. Twenty-four hours after training, the animals received bilateral intra-dorsal CA1 infusions of phosphorothioated Zif268 antisense oligonucleotides (ASO; 2 nmol/side), able to reduce basal Zif268 levels by ~50% within 90 min^26,27^ (Fig. 1a; t(2) = 7.033, p = 0.0196, one sample t test with theoretical mean = 100), or inactive scrambled ASO (sASO; 2 nmol/side). Ninety minutes after ASO or sASO injections, we re-exposed the animals to one of the familiar objects presented during training (object A) alongside a novel object (object C) to reactivate ORM and induce its hippocampus-dependent reconsolidation^7^. We assessed ORM retention 24 h later. To do that, we exposed the animals for 5 additional minutes to objects A, B or C together with novel object D. Rats that received sASO before ORM reactivation discriminated object D from objects A, B or C during the retention session (Fig. 1b), showing that they remembered objects A and B, both presented during the training session, and also that they acquired memory for object C during the reactivation session. Conversely, rats that received ASO did not discriminate objects A and C from object D (Test AD: t(16) = 3.963, p = 0.0011; Test CD: t(15) = 2.405, p = 0.0296, ASO vs sASO in unpaired t test), suggesting that Zif268 knockdown disrupted memory for object A and impaired formation of memory for object C. Memory for object B, which was present during the training session but absent during the reactivation session, was spared by ASO (Fig. 1b). ASO did not affect retention when given in dorsal CA1 6 h after ORM reactivation in the presence of a novel object (Fig. 1c) or when administered 90 min before either ORM reactivation in the presence of two familiar objects (Fig. 1d) or a 5 min-long exploration session of the training arena devoid of stimuli objects (Fig. 1e). Similarly, ASO had no effect on retention when administered 24 h after ORM training in the absence of reactivation (Fig. 1f) or when given 90 min before ORM reactivation but retention was tested 3 h instead of 24 h later (Fig. 1g). Pre-reactivation intra-CA1 infusion of the GABAa agonist muscimol (MUS; 0.1 µg/side), but not of ASO or sASO, disrupted ORM retrieval (Fig. 1h; F(3, 26) = 17.95, p < 0.0001; t(26) = 5.406, p < 0.001 for VEH vs MUS, t(26) = 5.468, p < 0.001 for sASO vs MUS, t(26) = 6.600, p < 0.001 for ASO vs MUS in Bonferroni’s multiple comparisons test after ANOVA).

**Figure 2:**
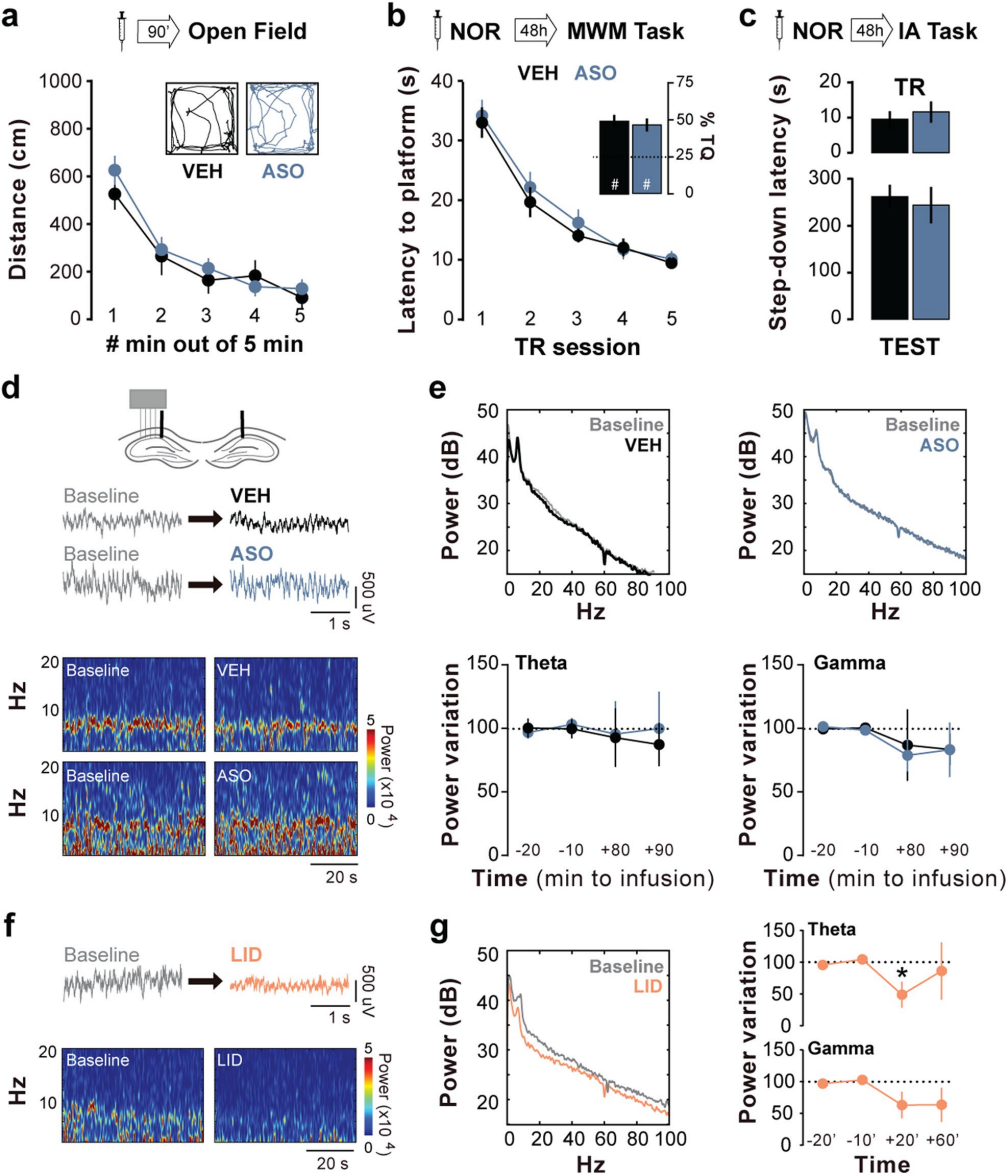
Zif268 knockdown does not disrupt hippocampus function. (**a**) Spontaneous locomotor activity during a 5′-long open field arena exploration session carried out 90′ after bilateral intra-dorsal CA1 infusions of vehicle (VEH; saline 0.9%) or Zif268 antisense oligonucleotides (ASO; 2 nmol/µl); n = 9 animals per group. (**b**) Animals that received ASO after ORM reactivation were trained in the spatial version of the water maze (MWM) 48 h later and compared to animals given VEH into dorsal CA1 30 min before the first MWM training session. Data show escape latency during MWM training (8 trials/session blocks) and % of time spent in target quadrant (TQ) during a 60 s-long probe test carried out 24 h after the last MWM training session; n = 9 animals per group. #p < 0.05 in one-sample Student’s t-test with theoretical mean = 25. (**c**) Animals that received ASO after ORM reactivation were trained in inhibitory avoidance (IA) 48 h later and compared to animals given VEH into dorsal CA1 30 min before IA training (TR). Data show step-down latency during TR and retention test (TEST) sessions; n = 8 animals per group. (**d**) Representative raw hippocampal LFP traces and spectrograms at different pre- (Baseline) and post-infusion time points from animals given intra-dorsal CA1 VEH or ASO. (**e**) Representative power spectrum density plots and mean theta (5–10 Hz) and gamma (35–100 Hz) power variation at different pre- or post-infusion time points from animals given VEH or ASO into dorsal CA1. (**f**) Representative raw hippocampal LFP traces and spectrograms at different pre- and post-infusion time points from animals given intra-CA1 lidocaine (LID; 4%) as a positive control. (**g**) Representative power spectrum density plots and mean theta and gamma power variation at different pre- and post-infusion time points from animals given intra-CA1 LID. Data are presented as mean ± SEM; n = 4 animals per group. *p < 0.05 in Bonferroni’s multiple-comparison test after one-way ANOVA.

We did not observe seizure-like episodes following ASO administration, which did not affect locomotor activity in an open field either (Fig. 2a). Animals rendered amnestic for ORM with ASO were later able to acquire a spatial preference (Fig. 2b) and a fear-motivated avoidance response (Fig. 2c) when trained in the water maze or the step-down inhibitory avoidance task, respectively. Spontaneous hippocampal oscillatory activity analyses indicated that intra-dorsal CA1 ASO did not modify on-going LFP in freely moving rats (Fig. 2d,e), unlike lidocaine, which was used as a positive control (Fig. 2f,g).

**Figure 3:**
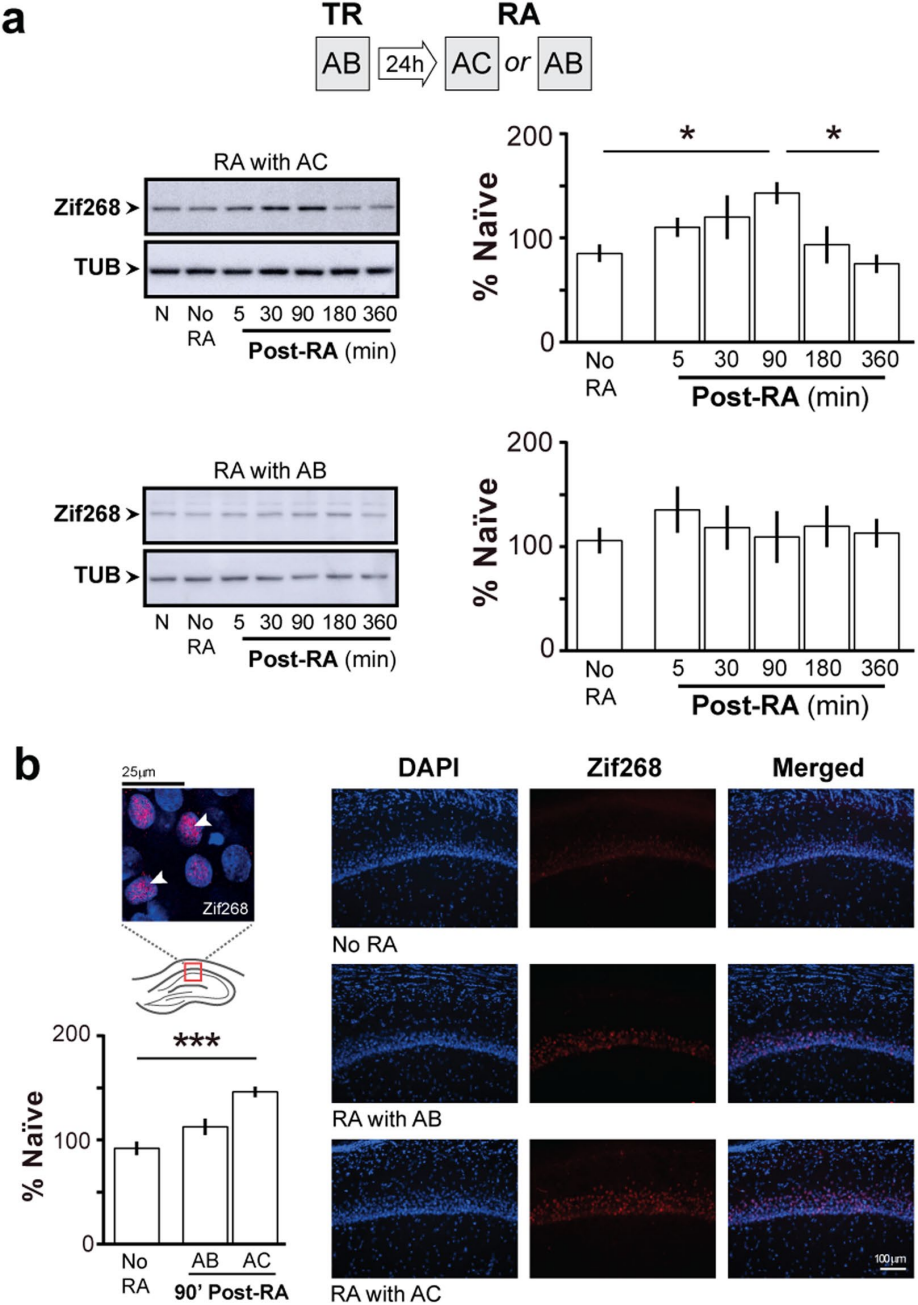
ORM reactivation in the presence of a novel object increases hippocampal Zif268. (**a**) Rats trained in NOR (TR) with objects A and B were submitted to a 5′-long ORM reactivation session (RA) in the presence of familiar object A and novel object C or familiar objects A and B 24 h post-training. At different times after RA, animals were killed by decapitation and the dorsal CA1 region dissected out and homogenized to determine Zif268 and β-tubulin (TUB) by immunoblot. A group of NOR trained animals not submitted to RA and killed 24 h post-training (No RA) was used as control; n = 4–5 animals per group. For full-length blots see Supplementary Figure S2 in Supplementary Information. (**b**) Rats were treated as in (**a)** but 90′ post-RA were transcardially perfused with PFA 4% and their brains removed and processed to determine Zif268 levels by immufluorescence. Representative images show Zif268 in dorsal CA1 (in red, counterstained with DAPI in blue); n = 5 animals per group. Data are presented as mean ± SEM. *p < 0.05, ***p < 0.001 in Bonferroni’s multiple-comparison test after one-way ANOVA.

Immunoblots of total homogenates from dorsal CA1 showed that ORM reactivation in the presence of a novel and a familiar object, but not in the presence of two familiar ones, increased Zif268 protein levels in a time-dependent manner, peaking 90 min post-reactivation and returning to pre-ORM reactivation control values 360 min after RA (Fig. 3a; F(5, 20) = 4.431, p = 0.0070; t(20) = 3.453, p < 0.05 for NoRA vs Post-RA 90, t(20) = 4.052, p < 0.01 for Post-RA 90 vs Post-RA 360 in Bonferroni’s multiple comparisons test after ANOVA). Immunofluorescence analyses confirmed that Zif268 expression augmented in dorsal CA1 only after ORM reactivation in the presence of a novel object (Fig. 3b; F(2, 12) = 17.30, p = 0.0003; t(12) = 5.826, p < 0.001 for NoRA vs AC, t(12) = 3.608, p < 0.05 AB vs AC in Bonferroni’s multiple comparisons test after ANOVA).

**Figure 4:**
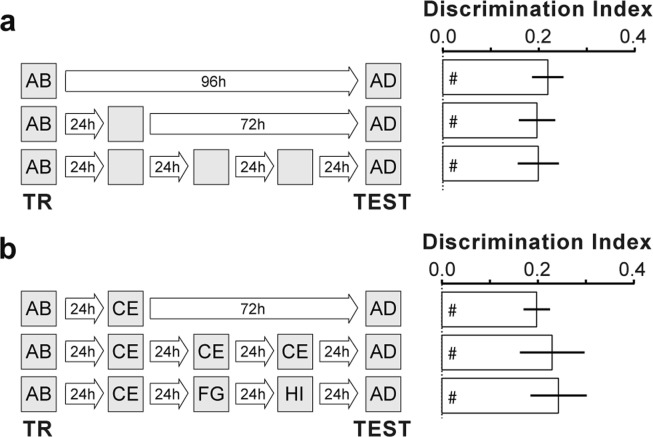
ORM is not susceptible to extinction. Rats trained in NOR (TR) using objects A and B were submitted to different post-training behavioural protocols. (**a**) One group of rats was tested for retention (TEST) in the presence of familiar object A and novel object D 96 h after TR. A second group was allowed to freely explore the training arena devoid of stimuli objects during 5′ 24 h after TR and tested 72 h later. A third group of animals was exposed to the training arena in the absence of stimuli objects during 5′ once daily for 3 consecutive days and TEST was carried out 24 h thereafter. (**b**) One group of rats was allowed to explore the training arena in the presence of novel objects C and E during 5′ 24 h after TR and tested 72 h later. A second group of animals explored the training arena in the presence of novel objects C and E during 5′ once daily for 3 consecutive days and tested for retention 24 h after the last exploration session. A third group was allowed to explore the training arena in the presence of a different pair of novel objects (CE/FG/HI) each day for 3 consecutive days and TEST was carried out 24 h after the last exploration session. Data (mean ± SEM) are presented as discrimination index during TEST; n = 9–10 animals per group. Dashed lines represent chance level. #p < 0.05 in one-sample Student’s t-test with theoretical mean = 0. Animals explored objects equally during TR. Total object exploration time did not differ between TR and TEST. For mean object exploration time and discrimination index values during TR see Supplementary Table S1 in Supplementary Information. For mean object exploration time values during TEST see Supplementary Table S3 in Supplementary Information. For mean object exploration time and discrimination index values during post-training exploration sessions see Supplementary Table S4 in Supplementary Information.

**Figure 5:**
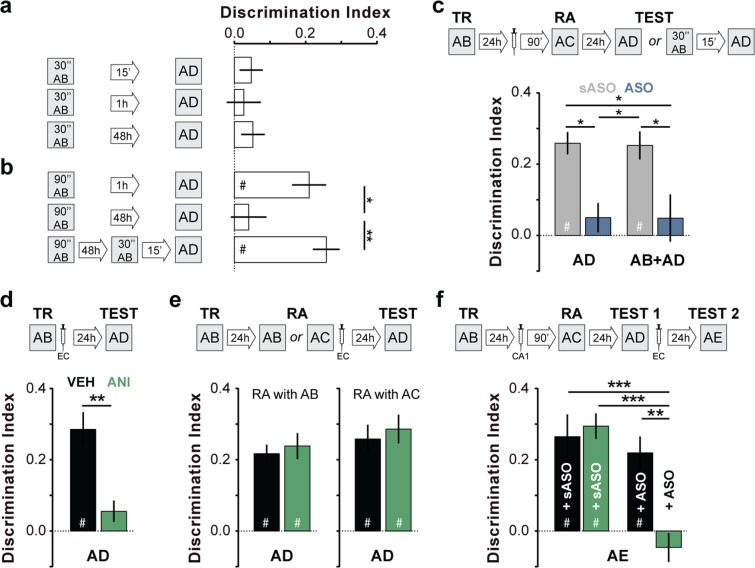
Zif268 knockdown during reconsolidation deletes ORM. (**a**) Rats were exposed to objects A and B for 30″ and tested for ORM 15′, 1 h or 48 h later in the presence of familiar object A and novel object D. (**b**) Rats were exposed to objects A and B for 90″ and either tested for ORM 1 h or 48 h later or re-exposed for 30″ to objects A and B 48 h post-training and tested 15′ later. (**c**) Rats trained in NOR (TR) with objects A and B received Zif268 antisense oligonucleotides (ASO; 2 nmol/µl) or scrambled ASO (sASO; 2 nmol/µl) into dorsal CA1 24 h post-training and 90′ later were submitted to a 5′-long ORM reactivation session (RA) in the presence of familiar object A and novel object C. Twenty-four hours later, animals were handled or re-exposed to objects A and B during 30″ and 15′ thereafter tested for ORM in the presence of familiar object A and novel object D. (**d**) Rats were trained as in **c** but immediately post-training received vehicle (VEH; saline 0.9%) or anisomycin (ANI, 160 µg/µl) in entorhinal cortex (EC). Retention was assessed 24 h later. (**e**) Rats were trained as in (**c**) and 24 h later submitted to RA in the presence of familiar objects A and B or familiar object A and novel object C. Immediately after RA, the animals received intra-EC infusions of VEH or ANI. Retention was assessed 24 h later. (**f**) Rats trained as in **c** received sASO or ASO into dorsal CA1 24 h post-training and 90′ later were submitted to RA in the presence of familiar object A and novel object C. Retention was assessed 24 h later (TEST 1) in the presence of familiar object A and novel object D. Immediately after TEST 1, rats received VEH or ANI into EC. ORM was tested 24 h later (TEST 2) in the presence of familiar object A and novel object E. Data (mean ± SEM) are presented as discrimination index during TEST; n = 8–10 animals per group. Data in panel 5 f are presented as discrimination index during TEST 2. For mean discrimination index values during TEST 1 see Supplementary Figure S3 in Supplementary Information. Dashed lines represent chance level. #p < 0.05 in one-sample Student’s t-test with theoretical mean = 0; *p < 0.05, **p < 0.01 and ***p < 0.001 in unpaired t-test or Bonferroni’s multiple-comparison test after two-way ANOVA. Animals explored objects equally during TR. Total object exploration time did not differ between TR, RA and TEST. For mean object exploration time and discrimination index values during TR see Supplementary Table S1 in Supplementary Information. For mean object exploration time and discrimination index values during RA see Supplementary Table S2 in Supplementary Information. For mean object exploration time values during TEST see Supplementary Table S3 in Supplementary Information.

Repeated or extended reactivation in the absence of the unconditioned stimulus induces extinction of conditioned memories. Extinction involves formation of a new memory trace that suppresses the behaviour driven by the original one without erasing it and is regarded as a process opposite to reconsolidation^28–31^. Recently, it was shown that a brief reminder session, unable to generate a lasting memory per se, reversed the amnesia caused by hippocampal Zif268 knockdown during contextual fear memory recall^25^. This indicates that the amnesic effect of Zif268 inhibition may be transient, and suggests that activation of this transcription factor prevents non-declarative memory extinction instead of promoting reconsolidation^25^. We found that neither repeated re-exposure to the training arena in the absence of stimuli objects (Fig. 4a) nor repeated re-exposure to the same pair of novel objects or exposure to a different pair of novel objects once daily for 3 consecutive days (Fig. 4b) had any effect on original ORM retention. In addition, a brief reminder session, too short to generate ORM by itself (Fig. 5a) but long enough to reverse the ORM deficit caused by the passage of time (Fig. 5b; F(2, 27) = 6.351, p = 0.0055; t(27) = 2.642, p < 0.05 for 1 h vs 48 h, t(27) = 3.393, p < 0.01 48 h vs 48 h + AB in Bonferroni’s multiple comparisons test after ANOVA), was unable to overturn the amnesia induced by intra-CA1 ASO (Fig. 5c; F(1, 36) = 19.79, p < 0.0001 for pharmacological treatment main effect; F(1, 36) = 0.0056, p = 0.9403 for reminder effect; F(1, 36) = 0.0029, p = 0.9573 for interaction; t(36) = 3.184, p < 0.05 for sASO/AD vs ASO/AD, t(36) = 3.199, p < 0.05 for sASO/AD vs ASO/AB + AD, t(36) = 3.092, p < 0.05 for sASO/AB + AD vs ASO/AD, t(36) = 3.107, p < 0.05 for sASO/AB + AD vs ASO/AB + AD in Bonferroni’s multiple comparisons test after ANOVA).

ORM consolidation, but not ORM reconsolidation, necessitates protein synthesis in the entorhinal cortex^10,32^ (EC; Fig. 5d,e; t(14) = 4.044, p = 0.0012, ANI vs VEH in unpaired t test). We found that animals made amnestic by pre-reactivation ASO administration reacquired the memory for the forgotten object A upon retraining. However, bilateral intra-EC infusion of the protein synthesis inhibitor anisomycin (ANI) right after ORM retraining abolished relearning as if ORM had to be consolidated again, further endorsing the notion that the amnesia induced by reactivation-targeted hippocampal Zif268 knockdown was not transient but permanent (Fig. 5f; F(1, 28) = 16.96, p = 0.0003 for intra-CA1 treatment main effect; F(1, 28) = 6.345, p = 0.0178 for intra-EC treatment main effect; F(1, 28) = 9.978, p = 0.0038 for interaction; t(28) = 4.693, p < 0.001 for sASO/VEH vs ASO/ANI, t(28) = 5.146, p < 0.001 for sASO/ANI vs ASO/ANI, t(28) = 4.015, p < 0.01 for ASO/VEH vs ASO/ANI in Bonferroni’s multiple comparisons test after ANOVA).

## Discussion

Ample evidence indicates that reactivation labilizes ORM, which then requires protein synthesis-dependent reconsolidation to persist^7,33,34^. In fact, several studies showed that ORM is susceptible to intracerebral administration of metabolic blockers around the time of retrieval^35–37^. In particular, the hippocampus is engaged in reconsolidation only when ORM reactivation happens simultaneously with presentation of a novel object. This suggests the hippocampus processes information pertaining to novelty detection during ORM retrieval, as it has been reported to do also during other memory processes^38–40^, and mediates the incorporation of that information into the reactivated recognition trace through reconsolidation^7,8,41,42^. In agreement with this hypothesis, we found that ASO hindered ORM when infused into dorsal CA1 before exposing animals to a novel and a familiar object, but had no effect on subsequent retention when ORM was reactivated in the presence of two familiar objects. The amnesic effect of ASO took more than three hours to develop, was time-dependent, specific for the familiar object present in the reactivation session, and did not happen in the absence of explicit ORM reactivation, indicating that it was caused by disruption of the active ORM trace and not by a deleterious effect on performance. The fact that ASO did not impair ORM retrieval, together with the observation that ORM reactivation increased hippocampal Zif268 expression only when it happened in the presence of a novel object, further support this last assertion.

Intracerebral administration of phosphorothioated oligonucleotides can be neurotoxic and decrease neuronal excitability even before protein synthesis inhibition becomes apparent^43,44^. Indeed, a single injection of ASO may cause a rapid and long-lasting reduction in hippocampal field potentials^45^. To overcome these drawbacks we utilized a dose of ASO able to diminish hippocampal Zif268 protein levels just by 50% and performed a series of control experiments showing that ASO did not alter spontaneous oscillatory activity and that its amnesic effect was not mimicked by an equal dose of sASO. Moreover, we found that the amnesic animals that received ASO before ORM reactivation were later able to consolidate and express two different hippocampus-dependent memories, confirming that knocking down Zif268 expression does not induce lasting detrimental consequences on hippocampus function.

Whether inhibition of reconsolidation causes permanent memory erasure or induces temporary retrieval failure is a question that remains unanswered^46^. A recent report on this matter showed that contextual fear conditioning (CFC) retrieval-targeted hippocampal Zif268 knockdown did not result in permanent amnesia but memory recovered after presentation of a reminder^25^. This led the authors to challenge the well-documented role of Zif268 in conditioned memory reconsolidation^22–24^, and to question the very existence of reconsolidation as a distinct mnemonic process by suggesting that the molecular events induced by brief non-reinforced retrieval, including those mediated by Zif268, would act to prevent untimely extinction instead. Regardless of possible alternative interpretations for these results^46,47^, it is necessary to emphasize that different from CFC, which is a nondeclarative memory prone to extinction^48–50^, ORM is an inextinguishable declarative memory (Fig. 4). Also, unlike it was reported for CFC, a reminder session did not reverse the amnesia that ASO caused on ORM (Fig. 5c). Therefore, although it may be possible to construe the effect of Zif268 knockdown on CFC as the result of uninhibited extinction, to do so for ORM would be unreasonable.

To evaluate the nature of the amnesia triggered by hippocampal Zif268 knockdown further, we capitalized on the fact that ORM consolidation, but not its reconsolidation, necessitates de novo protein synthesis in EC^32^. We hypothesized that if the amnesic effect caused by blocking reconsolidation with ASO resulted from ORM erasure then re-presentation of the forgotten object would recap ORM consolidation because the animals should have to form the memory for that object anew. We found that animals rendered amnestic with ASO infused into dorsal CA1 90 min before ORM reactivation reacquired memory for object A upon retraining. However, in agreement with the hypothesis that the amnesia provoked by ASO is permanent, inhibition of EC protein synthesis after ORM retraining abolished relearning, as if memory for object A had to be formed again.

In conclusion, we presented evidence that hippocampal Zif268 increases after ORM retrieval in the presence of a novel object, and showed that activity of this transcription factor is critical to update the reactivated recognition memory trace but not to recall it or keep it stored while dormant. We also demonstrated that the amnesia elicited by reconsolidation disruption with Zif268 antisense oligonucleotides is due to ORM erasure and not to ORM retrieval failure.

## Methods

### Animals

We used 3-month-old male Wistar rats (600) weighting 300–350 g. They were housed in groups of 5 per cage with food and water ad libitum and kept on a 12 h light on/off schedule at 23 °C. Experiments were performed during the light phase of the cycle. Researchers were blind to the animal’s treatment and behavioural condition. Procedures were performed in accordance with the USA National Institutes of Health Guidelines for Animal Care and approved by the local institutional ethical committee (Comissão de Ética no Uso de Animais - CEUA, UFRN. Protocol #022/2016).

### Injection cannulas and multi-electrode arrays implants

Anesthetized rats (ketamine 80 mg/kg; xylazine 10 mg/kg) were submitted to stereotaxic surgery to implant 22-gauge stainless steel guide cannulas aimed to the CA1 region of the dorsal hippocampus (AP, −4.2; LL, ± 3.0; DV, −3.0 in mm) and/or entorhinal cortex (AP, −6.8; LL, ± 5.0; DV, −8.1 in mm). Eight animals were also implanted with electrode arrays (50 μm tungsten wires coated with PFA arranged in a 2 × 8 250 μm-spaced configuration) aimed to dorsal CA1 (AP −3.6; LL + 2.4; DV −3.6 mm). Stereotaxic coordinates were taken from^51^. Ground screw electrodes were implanted in the parietal bone. After surgery, animals received subcutaneous meloxicam (0.2 mg/kg). Rats implanted with electrode arrays were housed individually. Experiments began not less than 10 days after surgery.

### Drugs and infusion procedures

Zif268 antisense (ASO; GGTAGTTGTCCATGGTGG′; 2 nmol/µl) and Zif268 scrambled antisense oligonucleotides (sASO; GTGTTCGGTAGGGTGTCA′; 2 nmol/µl) were phosphorothioated on the three terminal bases to avoid nuclease degradation. Oligonucleotides, anisomycin (160 µg/µl), lidocaine (4%) and muscimol (0.1 µg/µl) were dissolved upon arrival, aliquoted, stored at −20 °C and diluted to working concentration in sterile saline (0.9%) on the day of the experiment. For intra-cerebral drug delivery, infusion cannulas were fitted into the guides and infusions (1 μl/side at a rate of 0.5 μl/min) carried out using a Hamilton syringe coupled to an infusion pump. Infusion cannulas were left in place for at least one additional minute to minimize backflow. An equal volume of sterile saline served as vehicle control.

### Novel object recognition (NOR) task

Object recognition memory (ORM) was assessed using a novel object recognition task (NOR) based on the spontaneous behaviour of rats that preferentially explore a novel object when presented alongside a familiar one^52^. Before training in the NOR task, rats were allowed to freely explore the training box arena (a 60 × 60 × 60 cm open field) in the absence of stimuli objects for 20′/day during 4 days (Habituation sessions). One day later, rats were trained in NOR by allowing them to explore two different novel stimuli objects in the training arena for 5 min (Training session; TR). Twenty-four hours after TR, a 5´-long ORM reactivation session (RA) was carried out by re-exposing the animals to one of the familiar objects explored during TR alongside a novel object in the training arena. ORM retention was evaluated at different time-points after RA by re-exposing the animals to one of the familiar objects alongside a novel object D in the training arena during 5′ (TEST session). To avoid olfactory cues, the arena and the stimuli objects were cleaned with ethanol (50%) before each session. Stimuli objects were made of metal, glass, or glazed ceramic and had no significant innate preference for rats^10^. There were several copies of each object, which were used interchangeably. The role, identity and relative position of the stimuli objects were counterbalanced and randomly permuted. Object exploration was defined as sniffing and touching the stimuli objects with the muzzle and/or forepaws. Animal’s behaviour was recorded with digital video cameras placed above the training arenas. Video data were acquired at 30 frames/s and analysed using the ObjectScan system (CleverSys, RRID: SCR_017141). Discrimination index (DI) was calculated as: (Time exploring novel object – Time exploring familiar object) / Total object exploration time. Data from the entire 5´-long session were considered. DI can vary between −1 and + 1; positive DI scores represent preference for the novel object while DI scores close to zero (p > 0.05 in one-sample t test with theoretical mean = 0) represent no discrimination.

### Water maze task (MWM task)

The MWM task was conducted as described previously^53^. Briefly, a black circular pool (200-cm in diameter) was conceptually divided in four equal quadrants from which rats learned to escape onto a hidden platform placed 1.5 cm beneath the water surface. Water temperature was regulated at 24–25 °C. The maze was located in a room with several distal visual stimuli functioning as spatial cues. During five consecutive days, rats received eight training trials in which the hidden platform was kept in a constant location. Each trial initiated from a different starting location and consisted of a swim (60″ maximum) followed by a 30″ platform sit. One day after the last training session, memory retention was evaluated in a 60″-long probe trial carried out in the absence of the escape platform. Video data were acquired at 30 frames/s and analysed on-line using the TopScan system.

### Step-down inhibitory avoidance task (IA task)

The IA task was conducted as described previously^54^. Briefly, the training apparatus was a Plexiglas box (50 × 25 × 25 cm) with a platform (5 × 8 × 25 cm) on the left end of a series of bronze bars that made up the floor of the box. For training, animals were placed on the platform and when they stepped down to the grid received a 2″-long 0.4 mA-scrambled footshock. One day after training, we evaluated avoidance memory retention by placing the animals on the training box platform and measuring their latency to step down. The test session finished when the animals stepped down to the grid (footshock omitted) or after 300″.

### Immunoblotting

At different times after ORM reactivation (5′, 30′, 90′, 180′ or 360′), animals were killed by decapitation and the CA1 region of the dorsal hippocampus was dissected out and homogenized in ice-chilled homogenization buffer (10 mM Tris-HCl, pH 7.4, containing 0.32 M sucrose, 1 mM EDTA, 1 mM EGTA, 1 mM PMSF, 10 μg/ml aprotinin, 15 μg/ml leupeptin, 10 μg/ml bacitracin, 10 μg/ml pepstatin, 50 mM NaF, and 1 mM sodium orthovanadate). Protein concentration was determined using the BCA protein assay kit (Pierce; Cat#: 23235). SDS/PAGE was performed under reducing conditions and proteins were electro-transferred onto PVDF membranes. Blots were blocked for 2 h in TTBS (100 mM Tris-HCl, 0.9% NaCl, 0.1% Tween 20, pH 7.6), incubated overnight at 4 °C with anti-Zif268 (1:5,000; Santa Cruz Biotechnology; RRID: AB_2231020) or anti-cFos (1:6,000; Santa Cruz Biotechnology; RRID: AB_2106783) antibodies, washed with TTBS and incubated with HRP-coupled anti-IgG secondary antibody (1:200,000; Santa Cruz Biotechnology; RRID: AB_641181). West-Pico enhanced chemiluminescence kit (GE Healthcare; Cat#: RPN2108) was used to detect immunoreactivity. For loading control, blots were incubated with stripping buffer (Restore™ Western Blot Stripping Buffer) for 30 min at 37 °C, washed with TTBS and incubated overnight at 4 °C with anti-β-tubulin (1:40,000; Abcam; RRID: AB_2210370). Blots were then washed, incubated with HRP-coupled anti-IgG secondary antibody and immunoreactivity detected as described above. Blots were quantified using Amersham Imager 600 analysis software.

### Immufluorescence

Rats were deeply anesthetized with thiopental and transcardially perfused with paraformaldehyde (PFA; 4%; pH 7.2) 90 min after the reactivation session. Brains were removed, placed in PFA overnight, transferred to 30% sucrose for 48 h and sectioned with a cryostat. Free-floating coronal slices (50 μm) from the rostral-caudal extent of dorsal hippocampus were rinsed with PBS, incubated in PBST (0.2% Triton X-100) for 1 h and blocked with 10% NGS/PBST for 2 h at room temperature. Slices were incubated with anti-Zif268 (1:3,500) overnight at 4 °C, washed in PBST and incubated with anti-rabbit Alexa Fluor 594 (1:1,000; Invitrogen; RRID: AB_141359) for 2 h at room temperature. Slices were rinsed with PBS and counterstained with DAPI (1:1,000), mounted with Fluoromount-G (ThermoFisher) and stored at −20 °C until image acquisition. Images were acquired using a Leica DM5000 fluorescence microscope (10×/0.30na and 20×/0.50na objectives, filters A4 and TX2) and a CoolSNAP HQ2 CCD Camera in 16-bit greyscale. Images were analysed with Image-Pro Plus 7.0 software (Media Cybernetics; RRID: SCR_016879). Quantification of Zif268 immunoreactivity was performed in 4 sections per animal. Labelled nuclei were counted using automated custom macro scripts (roundness 1–5, perimeter 40–500, and background threshold 15) on original grayscale images. For visualization, pseudo-colour was applied (linear LUT covered full images) to images displayed in Fig. 3b.

### Electrophysiological recordings

To evaluate the effect of intra-CA1 ASO on spontaneous hippocampal oscillatory activity, we recorded local field potential (LFP) 30 min before (baseline) and ~70–100 min after VEH or ASO infusions using a Cerebus Neural Signal Processor system (Blackrock Microsystems). LFPs were acquired from freely moving rats placed in the recording cage. Signals were amplified, digitized, filtered at cut-off frequencies of 0.3 Hz and 250 Hz and sampled at 1 kHz. Data were analysed offline using built-in (Signal Processing Toolbox) and custom written routines in MATLAB (RRID: SCR_001622). The CA1 pyramidal cell layer was identified by stereotaxic coordinates and standard electrophysiological parameters, as described in^54^. The Welch periodogram method (3 s Hamming windows, 75% overlap) was used for power spectra computing. Power spectrum was calculated using sliding windows of 4 s and 2 s steps to construct spectrograms. We defined theta and gamma power as the average power in the frequency range of 5–10 Hz and 35–100 Hz, respectively. To determine the effect of intra-CA1 drug infusions on power variation, we calculated a hippocampal oscillatory activity post-infusion to pre-infusion power ratio considering 10 min-long epochs. Intra-CA1 infusion of 4% lidocaine was used as positive control.

### Data analysis

Statistical analyses were performed using GraphPad Prism 6 software (RRID: SCR_002798). Significance was set at p < 0.05. Novel object recognition data were analysed using one-sample t test with theoretical mean = 0, unpaired Student’s t test, or one-way/two-way ANOVA followed by Bonferroni’s multiple comparisons, as appropriated. MWM data were analysed using one-sample t test with theoretical mean = 25 or unpaired Student’s t test. IA data were analysed using unpaired Student’s t test (data from training) or two-tailed Mann–Whitney U test (300′′ ceiling imposed on retention test session latency). Immunoblots, immunofluorescence and electrophysiological data were analysed using ANOVA or repeated-measures ANOVA followed by Bonferroni’s multiple comparisons test, as appropriated

### Cannula and electrodes placement

Cannulas placement was verified using methylene-blue (4%; 1 μl/side), as described above. Dye spread was taken as indication of the diffusion of the drug previously infused. Data from animals with correct cannula implants (96%) were included in statistical analysis. To verify electrodes placement, anesthetized animals were transcardially perfused with PFA, the brains removed and left in 30% sucrose for 48 h before being cut into coronal sections (50 μm). Relevant slices were stained with cresyl-violet to confirm electrode tracks.

## Supplementary information

Supplementary Information

## Data Availability

Data are available upon request by contacting the corresponding author.
