# Supplementary material for: Recognition memory reconsolidation requires hippocampal Zif268
Source: Sci Rep. 2019 Nov 12;9:16620. doi: 10.1038/s41598-019-53005-8 (PMC6851087; doi:10.1038/s41598-019-53005-8)
Supplement: Supplementary file 1 — Supplementary Information [file 41598_2019_53005_MOESM1_ESM.pdf]

Recognition memory reconsolidation requires hippocampal Zif268

Supplementary Information

Maria Carolina Gonzalez[1,#], Janine I. Rossato[1,2,#], Andressa Radiske[1], Marina Pádua Reis[1], and Martín Cammarota[1,*]

[1]Memory Research Laboratory, Brain Institute, Federal University of Rio Grande do Norte, Av. Nascimento de Castro 2155, RN 59056-450, Natal, Brazil. [2] Departament of Physiology, Federal University of Rio Grande do Norte, Av. Sen. Salgado Filho, 3000, RN 59064-741, Natal, Brazil. * Correspondence should be addressed to Martín Cammarota at martin.cammarota@neuro.ufrn.br.

[#] M.C.G. and J.I.R. contributed equally to this work.

**Figure**	**Exploration time (s)**			**Discrimination Index**	**#*p***	***n***
	**Object (A)**	**Object (B)**	**Total**			
**1b**	30.45 ± 1.32	29.47 ± 1.23	59.92 ± 2.42	-0.016 ± 0.013	0.2258	53
**1c**	30.83 ± 1.63	30.77 ± 1.63	61.60 ± 3.09	0.001 ± 0.016	0.9384	48
**1d**	28.53 ± 1.75	28.85 ± 1.64	57.38 ± 3.16	0.009 ± 0.021	0.6752	20
**1e**	33.62 ± 2.20	33.97 ± 1.83	67.58 ± 3.75	0.012 ± 0.022	0.5821	18
**1f**	24.40 ± 1.63	24.90 ± 1.75	49.29 ± 3.28	0.008 ± 0.017	0.6218	18
**1g**	24.91 ± 1.79	26.90 ± 2.03	51.81 ± 3.54	0.035 ± 0.024	0.1838	14
**1h**	23.41 ± 0.99	23.79 ± 1.23	47.55 ± 2.04	0.002 ± 0.019	0.9177	30
**4a**	29.48 ± 1.78	28.50 ± 1.60	57.98 ± 3.22	-0.015 ± 0.017	0.3826	30
**4b**	27.81 ± 1.47	28.50 ± 1.76	56.31 ± 3.06	0.005 ± 0.018	0.7621	27
**5c**	30.95 ± 1.26	29.62 ± 1.24	60.58 ± 2.37	-0.023 ± 0.014	0.1078	40
**5d**	29.26 ± 3.28	29.98 ± 2.98	59.25 ± 3.06	0.161 ± 0.022	0.4822	16
**5e**	30.24 ± 3.53	31.01 ± 3.86	61.26 ± 7.28	0.007 ± 0.022	0.7305	16
	28.94 ± 2.83	27.22 ± 2.52	56.16 ± 5.16	-0.026 ± 0.021	0.2266	18
**5f**	25.18 ± 1.63	25.88 ± 1.66	51.06 ± 3.20	-0.011 ± 0.014	0.4229	32

Supplementary Table S1. Mean object exploration time and discrimination index (±SEM) during NOR training. Animals explored equally the two novel objects, #p in one-sample Student's t-test with theoretical mean = 0 for Discrimination Index data.

Figure	Exploration time (s)				Discrimination Index	#p	n
	Drug	Object (1)	Object (2)	Total			
1b	sASO	22.54 ± 1.65	34.73 ± 2.20	57.27 ± 3.16	0.211 ± 0.035	< 0.0001	27
	ASO	20.67 ± 1.37	28.84 ± 1.77	49.51 ± 2.82	0.165 ± 0.029	< 0.0001	26
1c	sASO	21.30 ± 1.40	34.42 ± 2.09	55.73 ± 3.19	0.232 ± 0.025	< 0.0001	24
	ASO	20.49 ± 1.53	35.57 ± 2.44	56.06 ± 3.67	0.271 ± 0.025	< 0.0001	24
1d	sASO	25.61 ± 1.67	25.26 ± 1.81	50.87 ± 3.21	-0.010 ± 0.031	0.7588	10
	ASO	24.25 ± 2.63	23.90 ± 2.53	48.15 ± 4.95	-0.011 ± 0.036	0.7683	10
1g	sASO	19.63 ± 2.99	29.79 ± 3.52	49.43 ± 6.38	0.214 ± 0.031	0.0005	7
	ASO	22.73 ± 2.53	34.45 ± 4.14	57.19 ± 6.51	0.202 ± 0.025	0.0002	7
5c	sASO	20.46 ± 1.18	33.77 ± 3.35	54.23 ± 4.05	0.199 ± 0.051	0.0011	20
	ASO	16.88 ± 1.58	29.08 ± 2.73	45.96 ± 4.04	0.264 ± 0.036	< 0.0001	20
5e	sASO	30.72 ± 2.40	32.83 ± 1.96	63.55 ± 3.73	0.036 ± 0.034	0.3260	8
	ASO	27.81 ± 4.10	27.46 ± 3.14	55.26 ± 7.05	0.010 ± 0.032	0.7660	8
	sASO	22.16 ± 3.59	35.34 ± 4.39	57.50 ± 7.80	0.254 ± 0.038	0.0002	9
	ASO	22.49 ± 1.73	39.69 ± 3.99	62.18 ± 4.89	0.258 ± 0.058	0.0023	9
5f	sASO	20.49 ± 1.94	29.77 ± 3.17	50.26 ± 4.59	-0.181 ± 0.039	0.0004	16
	ASO	17.18 ± 1.81	25.92 ± 2.78	43.10 ± 4.00	-0.196 ± 0.039	0.0001	16

Supplementary Table S2. Mean object exploration time and discrimination index (±SEM) during ORM reactivation. #p in one-sample Student's t-test with theoretical mean = 0 for Discrimination Index data. Total exploration time did not differ between sASO- and ASO-treated animals (p > 0.05 in unpaired t test).

Figure	Drug	Total Exploration time (s)	n
**1b**	sASO	51.45 ± 3.35	27
	ASO	52.62 ± 3.36	26
**1c**	sASO	52.04 ± 4.03	24
	ASO	51.93 ± 4.25	24
**1d**	sASO	49.83 ± 4.40	10
	ASO	47.66 ± 4.66	10
**1e**	sASO	53.22 ± 7.11	9
	ASO	54.18 ± 4.79	9
**1f**	sASO	45.47 ± 4.70	9
	ASO	48.74 ± 5.04	9
**1g**	sASO	47.46 ± 9.29	7
	ASO	43.66 ± 6.69	7
**1h**	VEH	47.04 ± 1.58	8
	MUS	44.09 ± 4.14	8
	sASO	41.15 ± 4.82	7
	ASO	47.45 ± 6.33	7
**4a**	-	54.49 ± 3.46	10
	-	64.44 ± 4.93	10
	-	59.49 ± 5.37	10
**4b**	-	66.39 ± 7.31	9
	-	67.22 ± 5.82	9
	-	62.82 ± 4.69	9
**5c**	sASO	54.15 ± 5.83	10
		54.38 ± 7.34	10
	ASO	54.27 ± 6.01	10
		45.16 ± 5.44	10
**5d**	VEH	58.63 ± 10.16	8
	ANI	46.44 ± 6.56	8
**5e**	VEH	46.71 ± 5.28	8
	ANI	50.48 ± 6.17	8
	VEH	40.72 ± 3.90	9
	ANI	48.38 ± 6.52	9
**5f TEST1**	sASO	52.98 ± 4.69	16
	ASO	42.05 ± 3.04	16
**5f TEST 2**	sASO/VEH	47.05 ± 7.17	8
	sASO/ANI	48.99 ± 2.66	8
	ASO/VEH	38.66 ± 5.20	8
	ASO/ANI	42.05 ± 6.16	8

Supplementary Table S3. Mean object exploration time (±SEM) during ORM retention test.

Exploration time (s)				Discrimination Index	#*p*	*n*
**Group**	**Object (1)**	**Object (2)**	**Total**			
**CE**	26.12 ± 2.16	28.09 ± 2.84	54.21 ± 4.69	0.024 ± 0.038	0.5400	9
**CE/CE/CE**	27.81 ± 2.37	29.20 ± 2.11	57.01 ± 4.34	0.030 ± 0.018	0.1418	9
	33.25 ± 2.88	35.98 ± 2.59	69.23 ± 5.28	0.044 ± 0.023	0.0920	
	29.40 ± 3.36	33.19 ± 3.07	62.58 ± 4.69	0.065 ± 0.063	0.3303	
**CE/FG/HI**	24.39 ± 2.57	29.09 ± 3.92	53.48 ± 5.90	0.076 ± 0.050	0.1672	9
	29.42 ± 1.70	34.40 ± 2.86	64.32 ± 4.06	0.077 ± 0.038	0.0776	
	30.33 ± 3.68	35.16 ± 2.61	65.49 ± 5.60	0.090 ± 0.048	0.0983	

Supplementary Table S4. Mean object exploration time and discrimination index (±SEM) during exposure to two novel objects (CE) or during repeated exposure to the same pair of novel objects for 3 consecutive days (CE/CE/CE) or during exposure to a different pair of novel objects once daily for 3 consecutive days (CE/FG/HI). #p in one-sample Student's t-test with theoretical mean = 0 for Discrimination Index data.

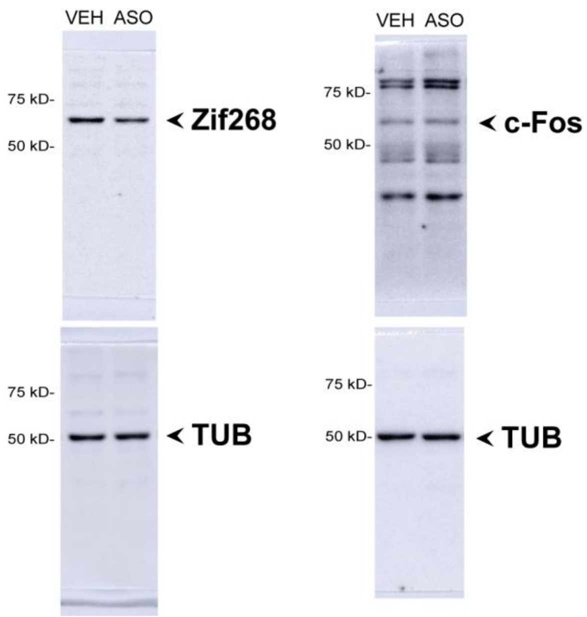

Supplementary Figure S1. Full-length blots presented in Fig 1.

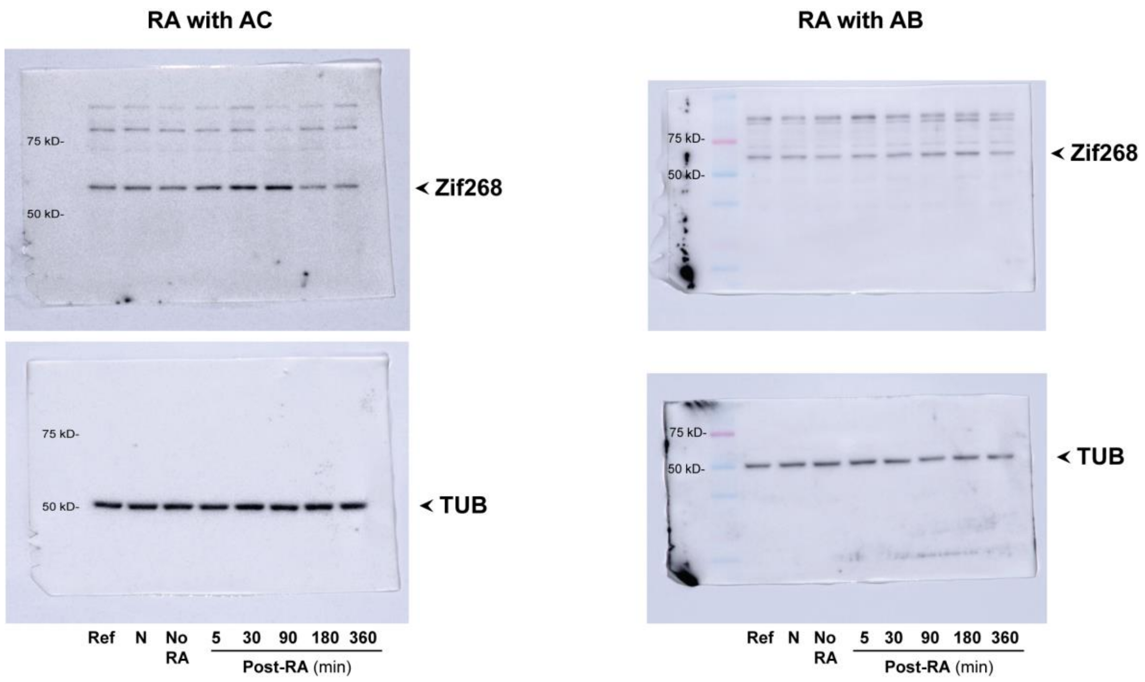

Supplementary Figure S2. Full-length blots presented in Fig 3.

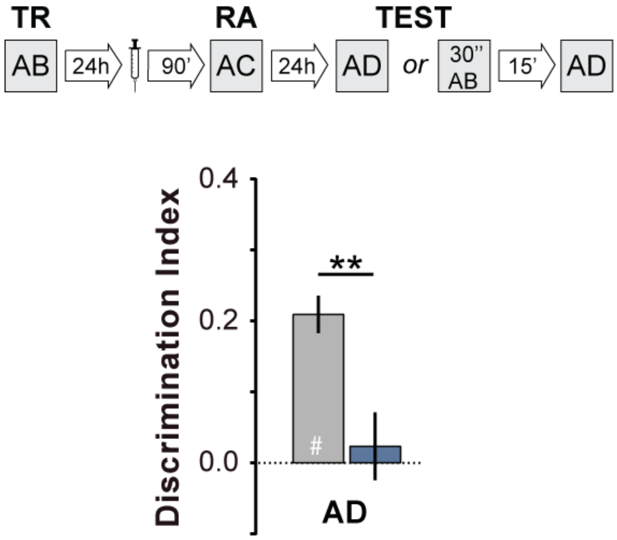

Supplementary Figure S3. Mean discrimination index values during TEST 1 from Fig 5f. Data (mean ± SEM) are presented as discrimination index during TEST 1; n = 16 animals per group. Dashed lines represent chance level. #$p < 0.05$ in one-sample Student's t-test with theoretical mean = 0; **$p < 0.01$ in unpaired t-test.
